# Habitat type modulates sharp body mass oscillations in cyclic common vole populations

**DOI:** 10.1038/s41598-024-62687-8

**Published:** 2024-05-26

**Authors:** Pedro P. Olea, Noelia de Diego, Jesús T. García, Javier Viñuela

**Affiliations:** 1 Terrestrial Ecology Group (TEG), Departamento de Ecología, Facultad de Ciencias, Universidad Autónoma de Madrid (UAM), 28049 Madrid, Spain; 2 Centro de Investigación en Biodiversidad y Cambio Global (CIBC-UAM), Universidad Autónoma de Madrid, 28049 Madrid, Spain; 3 Game and Wildlife Management Group, Institute for Game and Wildlife Research (IREC, UCLM-CSIC-JCCM), Ciudad Real, Spain

**Keywords:** Phenotypic variation, Rodents, Body size, *Microtus arvalis*, Biological trait, Phase-related changes, Multi-annual population fluctuations, Ecology, Zoology, Ecology, Environmental sciences

## Abstract

Cyclic rodent populations exhibit pronounced changes in body mass associated with the population cycle phase, long-known as Chitty effect. Although Chitty effect is a common epiphenomenon in both America and Europe, there is still incomplete evidence about the generality of these patterns across the entire range of most species. Moreover, despite decades of research, the underlying factors driving Chitty effect remains poorly understood. Here, we examined the influence of intrinsic and extrinsic factors that may underlie observed patterns in vole size variation in the Iberian common vole *Microtus arvalis asturianus*. We weighed and measured 2816 adult voles that were captured during 6 trapping periods. Vole numbers and body mass showed strong period- and phase-related variation both in females and males, demonstrating marked Chitty effect in the studied population. Body mass of adult males correlated with body length, evidencing that heavier males are also structurally larger. Statistical models showed that probability of occurrence of large-sized vole (> 37 g) was significantly more likely in reproductive males, during *increase* and *peak* phases, and it was modulated by habitat, with crop fields and field margins between crops showing an increased likelihood. We suggest an effect of the habitat on vole body mass mediated by predation.

## Introduction

Regular multi-annual population fluctuations exhibited by small rodents (mostly lemmings and voles) have attracted the attention of ecologists for almost a century^1–4^. This cyclic demography, first discovered in rodent populations at high northern latitudes, have been subsequently detected also in the southern hemisphere and at lower latitudes (e.g.^5,6^). However, despite extensive research on this widespread phenomenon, the mechanisms that generate population cycles is still a matter of debate (reviewed in^3,7^).

Cyclic populations exhibit periodic multiannual density fluctuations that are often described as a sequence of phases with different demographic traits (increase, peak, decrease and low phases) in which a peak occurs most often every 3–5 years^7^. It is noteworthy that cyclic rodent populations are normally coupled with phase-related changes in biological traits, which are known to vary in a systematic manner along the population cycle^3^, see also^8^ for non-cyclic species. For example, during the peak phase, traits such as survival and reproductive rates are reduced, whilst susceptibility to infections, aggressive behavior and body mass are increased^3^.

Phase-related changes in rodent body mass have attracted the most interest, as adults in the peak phase are conspicuously up to 20–30% heavier on average than in low phases^9^. This phenomenon, called the *Chitty effect*^10^, is nearly ubiquitous across cyclic vole and lemming populations in boreal and temperate climates of Europe and North America^4,9,11,12^, with few reported exceptions^13,14^. Although body size is considered the most important trait of animals because of its ecological consequences^15^, the true interest raised by this topic lies in the fact that, given the tight association between cycle phase and this phenotypic variation, understanding the Chitty effect could help to explain the enigma of population cycles (e.g.^9,16–19^; but see^8^).

Due to the many ecological implications of body size^20^, understanding the patterns and drivers of body size variation has been a central theme of ecology over the last decades (e.g.^15,21,22^). In small rodents, the debate on the relative importance of intrinsic (genetically-determined, maternal effects or changes in behavior or physiology) *versus* extrinsic (environment-related) factors in generating the observed fluctuations in body mass is still open^9,23,24^. There is, however, a noticeable lack of information in the occurrence of body size patterns in cyclic southern –Mediterranean– vole populations in Europe (but see^12^ for similar latitudes in America). Thereby, research on southern European populations is necessary to complete our knowledge about potential generality of those patterns across the entire vole range, especially regarding the reported latitudinal gradients in the degree of cyclicity (e.g. northern vole populations display cycles with larger amplitude and higher periodicity than their southern counterparts both in Fennoscandia and Japan^25–27^). These geographical variations in cyclicity seem to be particularly frequent in the Common vole (*Microtus arvalis*), showing a gradient from non-cyclic populations to cycles highly variable in amplitude and periodicity (reviewed in^28^). These variations in demography may fit latitudinal gradients or not^29,30^, but there is almost no published information about the *Chitty* effect in this species (but see^31^).

The Iberian common vole (*Microtus arvalis asturianus*) is now considered as a long-term isolated endemism of the Iberian Peninsula^32^, the southwestern edge of the species’ range, without recent genetic admixture with other European populations and with distinctive phenotypic traits, including a relatively large average body size as compared to nominal subspecies^33,34^. Cyclicity in the Iberian vole populations has been reported over four decades in a large population which has recently colonized an extensive agricultural area in NW Spain, where it shows high-density population outbreaks every 2–5 years, causing crop damages and spill-over of tularemia disease^6,35–37^. This pattern of recently emerging population cycles at the southernmost latitudes contrasts with the recent decrease in cyclicity recorded at northern latitudes^7,38^. To date, however, there has been no attempt to evaluate the existence of phenotype variations coupled with phase of cycle at the southern edge of the range, neither has there been any examination of factors potentially driving those variations in common voles. Monitoring the prevalence of individuals above a critical weight threshold might serve as an early indicator of a potential phase change in the population cycle, with significant implications for pest management strategies.

Here, we tested the hypothesis of the existence of *Chitty* effect in common vole populations at the southern edge of their distribution by analyzing phase-related changes in body mass in two high-amplitude cyclic populations of the Iberian common vole. We also tested the hypotheses of the influence of intrinsic (reproductive status) and extrinsic (site, habitat and vegetation features) factors driving observed patterns in vole size variation. We report, for the first time, the existence of the *Chitty* effect in cyclic populations in the SW range edge of the species and its modulation by habitat.

## Materials and methods

### Study area

**Figure 1:**
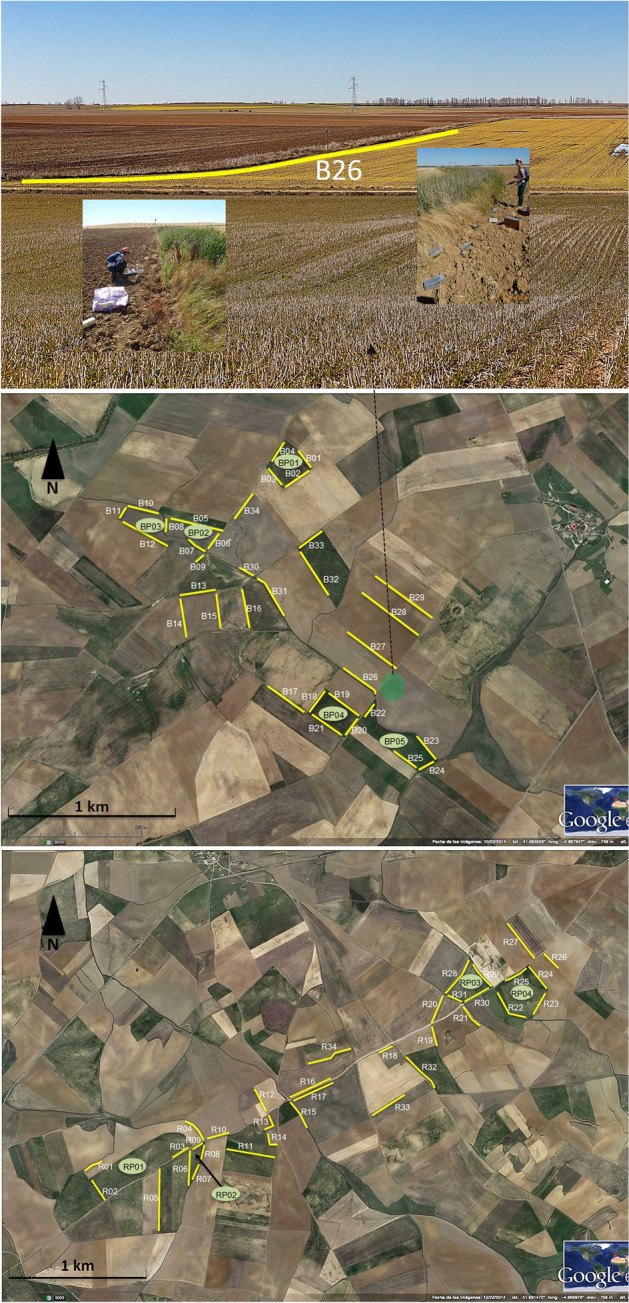
Satellite images of the study areas (*Boada* above, *Revilla* below) with the sampling locations; yellow lines are margins sampled, green polygons show crop fields sampled. Above: detail of the agricultural habitat in the study area (the image shows the surrounding area of the field margin sampled B26, yellow line); inset images: details of the placement and removal of the traps (at the field margin B26, July 2015). Photos by Pedro P. Olea.

The study was conducted in two sampling areas located in the localities of Boada de Campos (41° 59’ N, 4° 54’ W) and Revilla de Campos (41° 60’ N, 4° 42’W; Palencia province, Castilla y León region, NW Spain), about 14 km apart from each other. In both localities, called *Boada* and *Revilla* hereafter, the landscape is dominated by highly deforested dryland farming plains (Fig. 1) in which high incidence of common vole outbreak episodes occur frequently (every 2–3 years), with the highest amplitude and reaching the maximal densities at peak phase recorded in Spain (Fig. S1)^6,35,36,39,40^. The climate is Mediterranean, with an oceanic and high continental influence; winters are long and cold, and summers are hot and dry (annual mean Tª: 11.7 °C; January mean Tª: 3.5 °C; July mean Tª: 21 °C; annual precipitation: $\sim$ 400 mm (litres)/m^2^; Atlas Agroclimático de Castilla y León^36,41^). The main crops in the study area are winter cereals, mainly barley (*Hordeum vulgare*) and wheat (*Triticum spp*.), and alfalfa (*Medicago sativa*). Alfalfa is often cultivated by dry farming, but irrigation may also be used near irrigation channels. In this agricultural landscape mosaic, natural vegetation cover is scarce, almost exclusively dominated by annual herbs and grasses, with scarce and dispersed shrubs, and mainly restricted to crop field edges^40^. The areas temporally more stable are field margins, fallows or uncultivated areas, and multi-annual herbaceous crops, such as alfalfa, the optimal habitat in this agrarian region^42^. Boada area was included in a program promoting biological control of common voles by providing 100 nest-boxes for raptors (Common kestrel *Falco tinnunculus* and Barn owl *Tyto alba*), while no boxes were provided in Revilla^46^. This potential differential effect of aerial predation on our vole population was expectedly conveyed by the variable locality (see below, Table 2).

### Vole sampling

A longitudinal sampling was conducted in crop fields optimal for voles (alfalfa fields) and their margins, as well as in margins from other crops (mainly cereal and legume crops) (see^40^) (Fig. 1). The study was initially designed to study how landscape affect vole dispersal and ultimately gene flow in highly fragmented agricultural landscapes. Because of this, the sampling design was optimized to maximize captures in stable substrates (field margins, as potential corridors for dispersal, and alfalfa fields) within each study area, to the detriment of a more random design (e.g. trapping sites randomly distributed across the landscape). Vole populations were live-sampled using unbaited Sherman^®^ traps at the two study sites (Boada and Revilla), which were chosen as representative of the dominant regional landscape, and selected for their similarity in terms of landscape, and because the distance between them (14 km) ensured spatial independence. In each site, 5 (Boada) or 4 (Revilla) alfalfa fields were sampled, using 5 trapping points of 10 traps each, in each sampled field (totaling 250 traps in Boada and 200 in Revilla)^43^ (Fig. 1). Each trapping point tried to sample individuals on a different active colony, placing traps facing burrows with recent signs of activity (digging, clipping, droppings), or in the pathways to them. Voles were also sampled in field margins in each locality (n = 98 field margins in Boada and n = 44 in Revilla), with 5 trapping points (10 traps each) per field margin (50 traps/field margin). The same crop fields (n = 9) and the same field margins (n = 60) were repeatedly sampled over a 3 year period (2015–2017) in the two study areas (Fig. 1).

**Figure 2:**
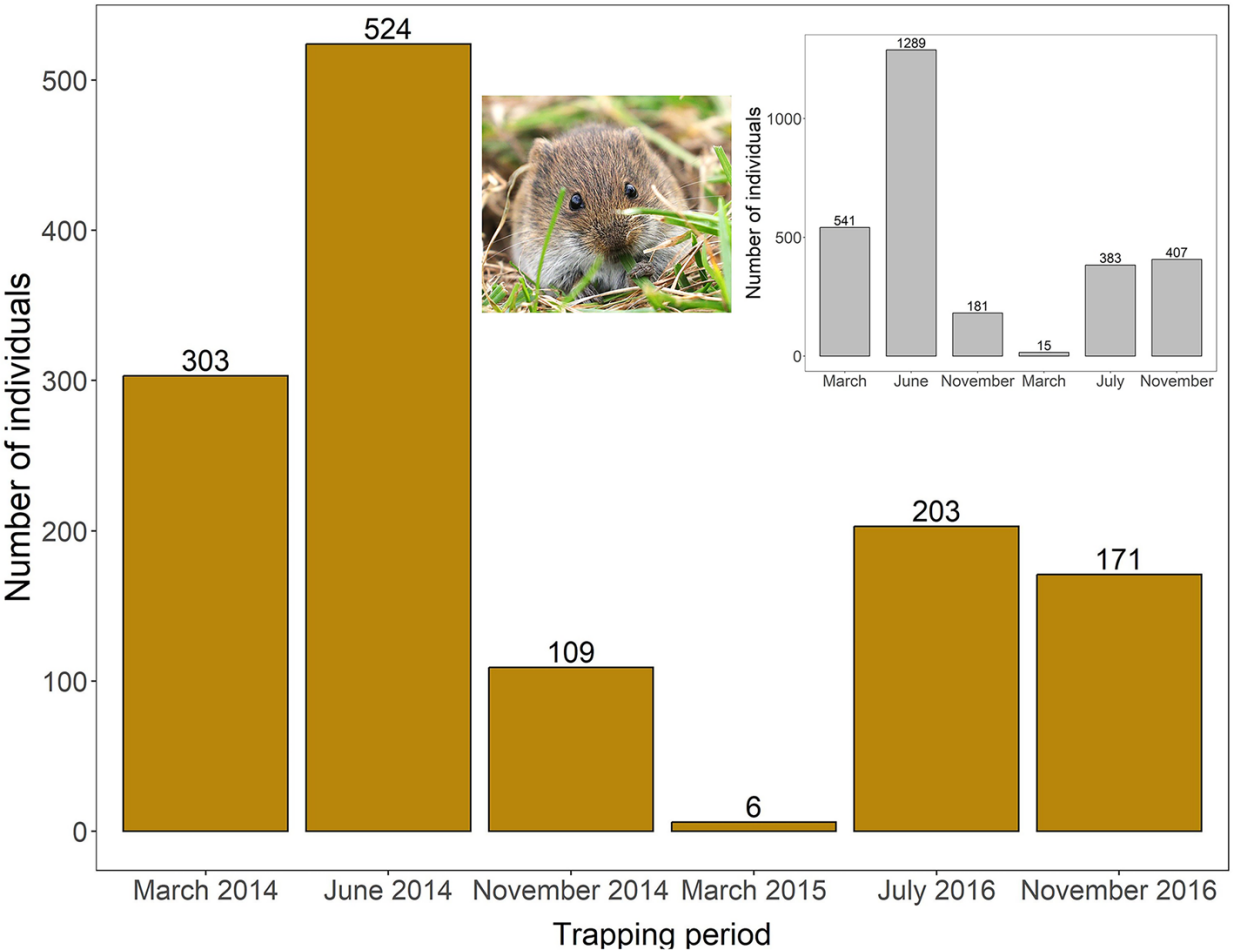
Adult common voles captured in each trapping period from March 2014 until November 2016 in the study area. Sampling effort was similar in each period (3181–3776 traps-nights/period). Total numbers of sexually mature individuals (males and females) are displayed in the inset, while only males are represented in the main plot. The temporal pattern of vole trapping was similar for total adult population and the male-only population. Values above the bars represent the number of voles captured. Photo by Rainer Löter.

For monitoring the cycle, voles were trapped seven times (1–3 times per year) depending on the cycle phase. The population was trapped in late October-early November (autumn: after the breeding season), late March (spring: early breeding season) and June-July (summer: late breeding season), coinciding closely with important phenological changes in agrarian landscapes (crop sowing, growing and harvesting, respectively) (Fig. 2). Due to the small vole population size reached in spring 2015 (only 14 individuals captured considering both study sites), sampling was halted until summer 2016, when vole density increased again, to guarantee enough captures. In December 2017, we did not get any vole capture (Fig. S1), and thus discarded this trapping period from the analysis. The length of each trapping session was of four consecutive days, two days per locality. All traps were opened in the late afternoon and checked the next morning (traps were in use for an average of 12 h period). Sampling effort was similar in both study sites, with a total of 3200–3700 traps/night for each trapping session. We georeferenced all trapping points in the field with a GPS device and marked with small flags. Each flag had a unique code to associate the captures to each trapping point. Capture rates per 100 traps/night were calculated in each trapping period, both for the whole adult population and only for males (see below for more details).

We took several measures of each captured vole: (i) body mass (0.01 g), (ii) body length (mm) measured as the distance between the tip of the snout and the base of the tail, (iii) sex, identified by visual examination of the external genitalia, and (iv) testes position in males, classified as scrotal or non-scrotal^44,45^. Capture rate for adult common voles in each trapping period was estimated dividing the number of adult voles trapped by the number of traps set, excluding closed traps with no capture.

Some voles were humanely euthanized via cervical dislocation without anesthesia. The animal collection and use was in accordance with all the relevant guidelines. All procedures were approved by the University of Castilla-La Mancha’s Committee on Ethics and Animal Experimentation (CEEA, UCLM, Spain; reference number PR20170201), were within the guidelines of the European and Spanish and policy for animal protection and experimentation and in compliance with the ARRIVE guidelines. We also had permits for trapping and manipulation of voles (EP-CYL/31/2013 and EP-CYL/418/2016).

### Environmental variables

Field margins were classified into three categories: (i) adjacent to streams or irrigation channels, (ii) adjacent to dirt track or road ditches (culverts hereafter), or (iii) adjacent to other crop fields (between-crop margin). In addition, vegetation was characterized in each trapping point within a 5 m radius area around the central point of the area occupied by traps. Average height of vegetation was measured in centimeters, and vegetation cover was estimated visually and expressed as a percentage, following^40^ (see also this paper for additional details).

### Data analysis

To avoid differences in age-related body mass, only data from adult common voles have been used. Therefore, juvenile voles were excluded from the dataset, considering as such those voles below 15 g^46^, if testes were not descended^44,45^. In addition, by working exclusively with adult voles we avoided bias in mean population weight due to changes in juvenile recruitment, associated with greater reproduction rates in spring and summer than in fall and winter^47,48^. Females were also excluded, as pregnancy is difficult to detect at early stages, and thus including females would introduce biases in the analysis of body mass patterns^44,45,47,48^ (but see Fig. S2).

#### Population level

We assessed patterns of body mass variation at population level by using frequency distribution histograms, built with the ggplot2 package^49^ of R. To describe differences in body mass of each trapping period, basic descriptive statistics have been used: mean, percentiles, the median as 50th percentile, and coefficient of variation^7^. Coefficient of variation allows to describe the variability of a sample in comparison to its mean, and thus, it is useful when comparing between populations with different means. It was calculated for each trapping period dividing the standard deviation by the mean and expressed in percentage (s/$\overline{x}$ ∙ 100). For each body mass frequency distribution, Fisher asymmetry coefficient (skewness) and kurtosis were calculated. To determine differences in the body mass frequency distributions between trapping periods, we used Kolmogorov–Smirnov tests. To assess statistical differences in average body mass among trapping periods, we used a Kruskal–Wallis test; a Dunn's test was later applied as a post hoc analysis to determine which trapping periods were different to each other^50^.

In cyclic vole populations, the presence of heaviest individuals in high-density phases has been described in other studies^11,51,52^. Because the presence of these heavy individuals in the population is a signal of the Chitty effect, we determined the number of heavy individuals in the vole population for each period. To do this, we calculated the 75th percentile of male body mass (body mass 75th = 37 g; N = 330) from the total males captured in the six trapping periods (N = 1316). These body mass threshold values included individuals about 30% heavier than the overall adult male mean of the total adult population (28.6 g) and about 50% heavier than the highest body mass of adult males captured in low population phase (25 g) (Table 1). Therefore, we have been conservative in order to apply the criteria of Chitty effect in our population.

**Table 1:** Descriptive statistics of body mass of adult male voles for each trapping period; “aggregate” refers to trapping periods pooled.

Adult males body mass (g)													
	Percentile									mean	Coefficient variation (%)	Skewness	Kurtosis
	0%	5%	25%	50%	75%	90%	95%	99%	100%				
**Aggregate**	15.01	16.27	19.64	26.03	37.04	43.74	46.83	51.44	60.78	28.63	35.95	0.55	− 0.87
March 2014	15.09	18.89	29.24	35.43	40.00	45.09	47.92	50.81	53.83	34.57	23.98	− 0.24	− 0.23
June 2014	15.05	15.97	18.54	21.90	36.16	44.52	47.76	53.31	60.78	27.00	40.56	0.91	− 0.54
November 2014	15.34	15.96	17.18	18.44	20.14	22.08	23.51	28.96	39.08	19.13	16.47	2.81	13.57
March 2015	16.61	17.20	19.86	22.59	24.04	24.80	24.95	25.07	25.10	21.73	15.17	− 0.44	− 1.68
July 2016	15.93	17.04	25.36	32.93	34.96	39.56	44.52	47.01	54.36	32.56	29.52	0.07	− 0.83
November 2016	15.01	16.69	19.84	22.92	27.93	34.83	39.26	43.24	45.80	24.72	27.85	1.01	0.30

#### Individual level

**Figure 3:**
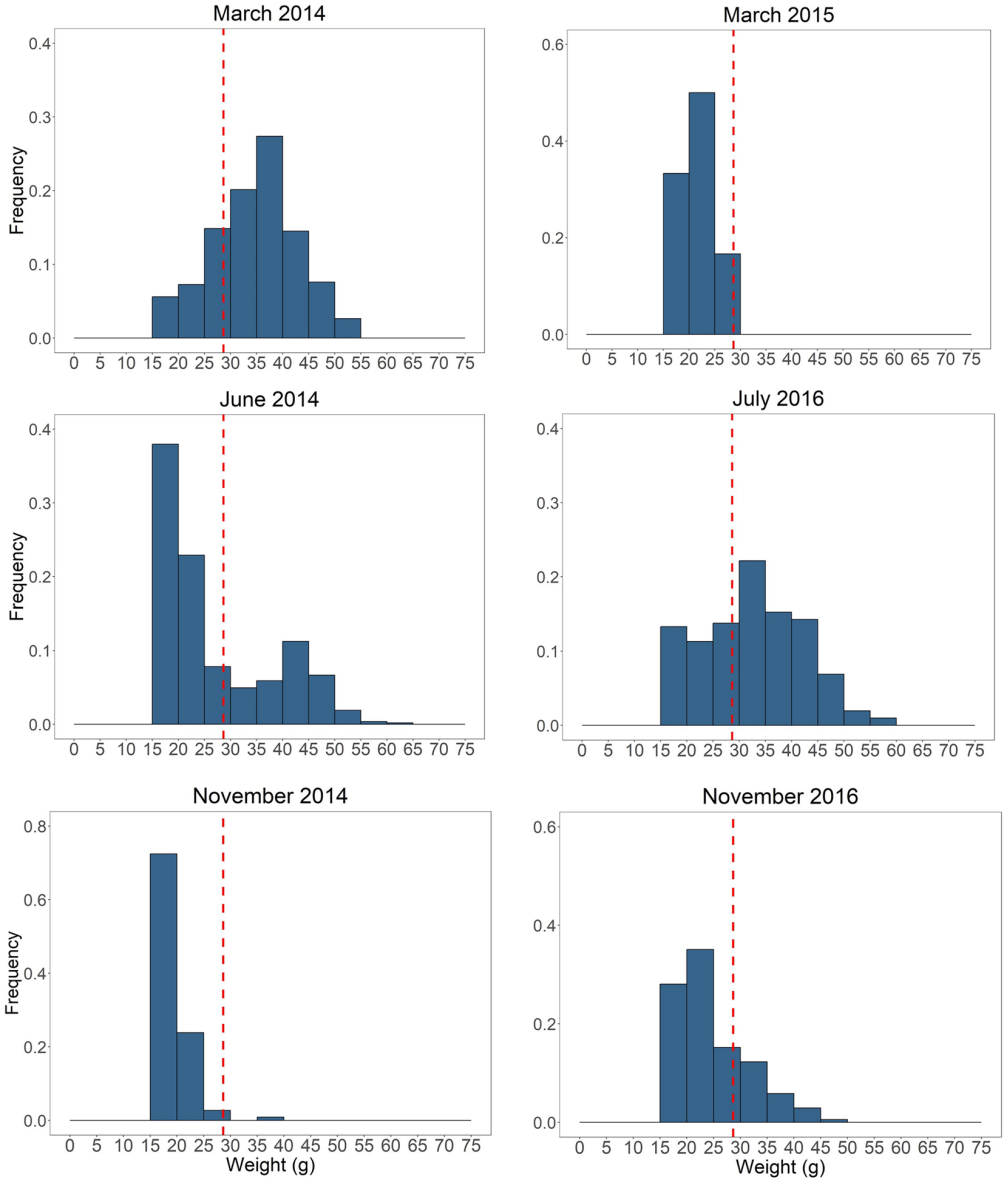
Frequency distribution histograms of body mass of adult male voles trapped in each period. Dash line represents mean body mass of the aggregated sample (28.63 g).

We analyzed factors explaining the probability that a given vole has a weight above the 75th percentile of total population body mass ($\ge$ 37 g) using binomial Generalized Linear Mixed Models (GLMMs). Thus, our response variables were whether or not the captured voles had body mass above 37 g ($\ge$ 75th: *N* = 330 voles; < 75th: *N* = 986). The explanatory variables considered were reproductive status of the individual (two levels: scrotal and non-scrotal testes), trapping period (six levels: March, June and November 2014, March 2015, and July and November 2016), sampling locality (two levels, Boada and Revilla), vegetation height and cover, measured following Planillo et al.^40^ (Table 2). Habitat type where vole was captured was included either as a two-level variable (arable fields *vs* field margins) or as a four-level variable (arable fields, between-crop margins, culverts and field margins adjacent to streams and irrigation channels). These two habitat variables were included in separate models. Because voles captured in the same field or margin cannot be considered independent from each other, we included in the models the crop field or margin ID where the vole was captured as a random factor. Moreover, according to the number of vole captures (Fig. 2**, **Fig. S1), we assigned trapping periods to phases of the population cycle: increase (March 2014), peak (June 2014, July 2016), decrease (November 2014, November 2016), low (March 2015). The 2016 demographic cycle appeared to be slightly delayed compared to 2014 (Fig. 2, Fig. 3). Since we were not entirely certain whether we conducted sampling during the peak phase in July 2016, we constructed an alternative variable in which the trapping period of July 2016 was designated as increasing phase. The variables cycle phases and trapping periods were included in separate GLMM models.

**Table 2:** Description of the explanatory variables considered in the study on vole body mass.

Variable	Code	Description
Trapping period	Period	Period when the sampling was done, March 2014, June 2014, November 2014, March 2015, July 2016, November 2016
Cycle phase	Phase	Cycle phase of the population when sampling was done: increase, peak, decrease and low
Vegetation cover	Veg.cover	Cover of vegetation at each trapping point in % (range 0–100%, total average 50.1%)
Vegetation height	Veg.height	Average height of vegetation at each trapping point, in cm (range 0–200 cm, total average 21.9 cm)
Locality	Locality	Locality where sampling was done (Boada, Revilla)
Habitat type	Margin.type	Crop field; margin next to stream, to path/ditch or between crops
Testes position in vole males	Reproductive.status	Classified as scrotal or non-scrotal
ID of margin or crop field	id	Identity of each margin or crop field sampled

We included variable interactions in the models to account for potential different effects of the explanatory variables during the different sampling periods (or population phases). Because none of the interactions were significant, we removed them from the full model to avoid an overly complex fitted model. We used the full model because we were interested in vole responses to the variables considered, rather than obtaining a simplified model. Nonetheless, model selection tables and effects of the AICc-based top ranking model are also provided in the supplementary information for comparison purposes (Table S2). The fixed part of the full model tested consisted of (see description of the variables in Table 2):

probability vole body mass $\ge$ 37 g ∼ sampling.periods/cycle.phases + reproductive.status + habitat.type + veg.cover + veg.height + locality.

GLMMs used z-test to assess the significance of model coefficients. Additionally, we employed the likelihood ratio test (LRT) to examine the effect of each variable included in the GLMM. The LRT compares the log likelihoods of the two models: the more complex model (with the variable included) versus the reduced model (without the variable included), testing whether the difference is statistically significant by using Chi-square test.

All statistical analyses were done in R 4.2.1 and 4.3.1^53^. GLMMs were run using the package lme4^54^. R^2^ values for the models were obtained using Nakagawa R^2^ for mixed models implemented in package MuMIn^55^. This same package was used for performing model selection, which was performed by generating subsets of the full model with all possible combinations of variables.

### Ethics approval

All handling procedures were approved by the UCLM Ethics Committee (reference number CEEA: PR20170201) and in accordance with the Spanish and European policy for animal protection and experimentation.

## Results

### Number of voles and phases of population cycle

A total of 2816 adult common voles were captured during the 6 trapping periods. While the sampling effort was comparable between trapping periods, the number of individuals captured varied greatly (Fig. 2). In the 2014–2015 period, the population exhibited a complete population cycle (increase, peak, decrease and low phases)^40^, as previously reported at larger spatial scale (Fig. S1)^6^. Population increased in spring (N = 515 in March 2014, capture rate, CR = 17.1 individuals per 100 trap nights), peaked in summer (N = 1,289 in June 14, CR = 38.5) and collapsed during the autumn (N = 120 in November, CR = 4.9), continuing the population decline through the winter to reach the minimum in the next spring (March 2015, N = 15, CR = 0.6) (Fig. 2). Number of adult voles trapped through 2016 did not follow the same pattern. Unlike 2014, results were similar between summer and fall trapping periods (July 2016, N = 383, CR = 10.3; November 2016, N = 407, CR = 11.7).

Number of males trapped (N = 1316) followed a similar pattern to total adults, peaking in June 2014 and collapsing in March 2015 (Fig. 2). In November 2016, the number of males trapped was slightly lower (N = 171, CR = 4.9) than in July 2016 (N = 203, CR = 5.4) (Fig. 2).

### Body mass frequency distributions and cycle phases

There were marked changes in the shape of the body mass frequency distributions between trapping periods (i.e. different population cycle phases) (Fig. 3). In the increase phase (March 2014) the body mass fitted well a gaussian distribution ranging from 15 to 54 g, whereas in the peak phase (June 2014) the range of body mass was broader (up to 61 g) and bimodal, with the second (minor) mode entirely corresponding to heavy males (> 29 g, above the mean, Table 1). Noteworthy, the population fraction of heavier adult males (about > 29 g) disappeared from the population in both the decrease and collapse phases (November 2014 and March 2015 respectively) resulting in leptokurtic distribution shapes and narrower range of body mass (Fig. 3). In the following population cycle of 2016, the large-size population fraction (> 37 g, the 75th percentile) was recovered, and distribution shapes of July and November 2016 were similar to both increase and decrease phases of 2014, respectively. Body mass frequency distributions were significantly different among cycle phases, except for March 2015 compared with June 2014 (K-S test, *D* = 0.389, p = 0.327), and with November 2016 (K-S test, *D* = 0.363, *p* = 0.431), likely due to the small sample size in the low-phase of March 2015.

### Means and percentiles of vole body mass

**Figure 4:**
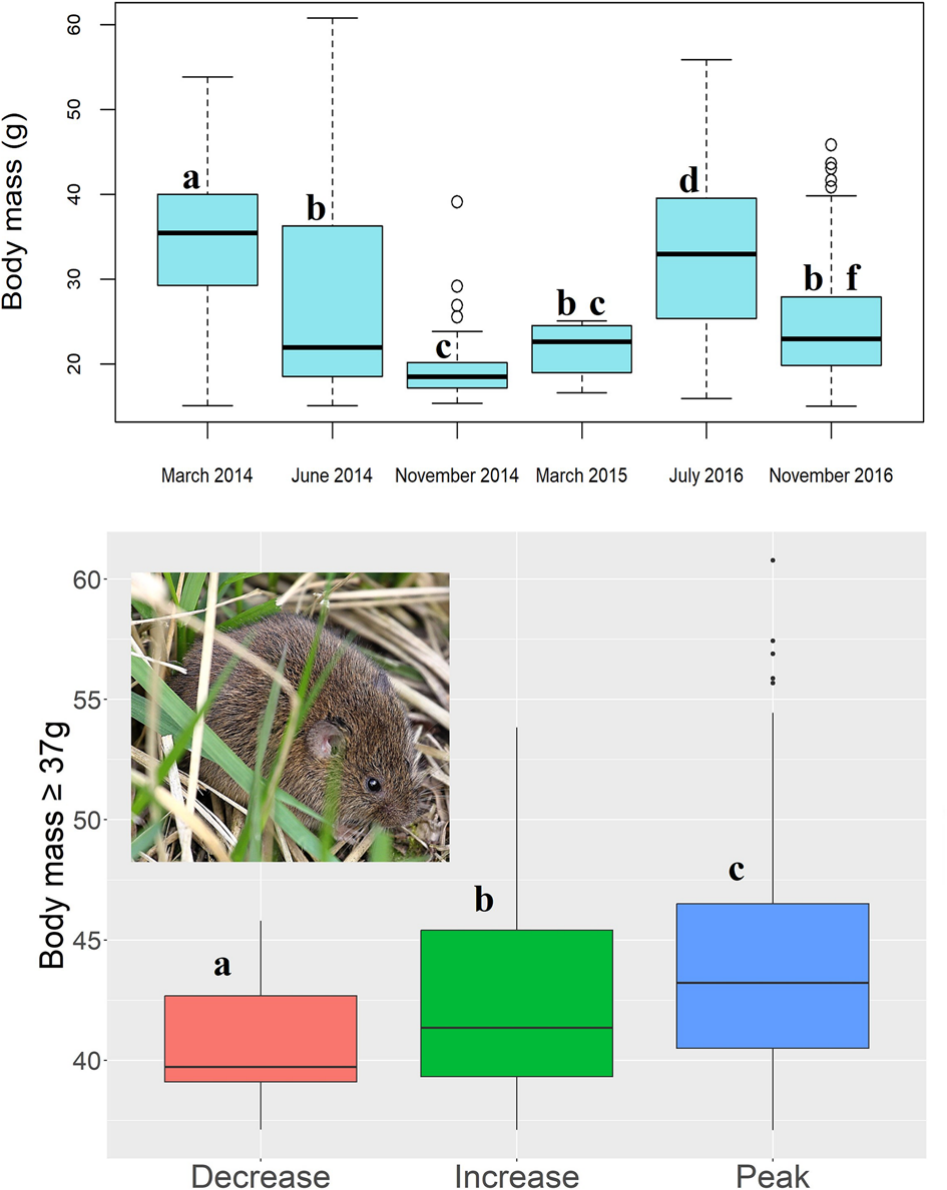
Boxplot for body mass of adult male voles trapped in each period (above) and for body mass $\ge$ 37 g according to the cycle phases (below). In low phase there was not voles $\ge$ 37 g. Boxes represent the interquartile range (IQR; the 25th and 75th percentiles), and median is depicted as a black line. The length of the whiskers shows values from the 75th percentile up to 1.5 × IQR (upper whisker) and from the 25th percentile down to − 1.5 × IQR (lower wisker); outliers are shown as dots above the whiskers. Letters correspond to statistical differences in body mass between trapping periods, as shown by the post-hoc Dunn test after performing the Kruskal–Wallis test; different letters denote statistical differences, same letters no statistical differences. Photo by Eloy Revilla.

In June 2014 (peak phase), the average body mass for adult males (27.0 g) decreased relative to March 2014 (34.6 g, increase phase), but the percentiles $\ge$ 90th were still comparable or larger than in March 2014 (45.1–53.8 *vs* 44.5–60.8 g) (Table 1**; **Fig. 4). In November 2014 (decrease phase), the mean weight dropped sharply to 19.1 g and no males over 40 g were trapped. The only six adult males trapped in March 2015 (when population collapsed, low phase) were small (all $\le$ 25 g) weighing 21.7 g on average. In the following population cycle of 2016, the mean body mass in July 2016 (peak phase) increased again to 32.6 g and decreased in November 2016 (24.7 g) (Table 1 and Fig. 4).

We selected 330 large-sized voles ($\ge$ 75^th^ or $\ge$ 37 g) for further analyses, representing 25% of the total male population captured. Percentages of large-sized voles over time were distributed as follows: March 2014 (increase phase; 38%), June 2014 (peak; 24%), November 2014 (decrease; 0.9%), March 2015 (low; 0%), July 2016 (peak; 35%) and November 2016 (decrease; 7.6%). Medians of body mass $\ge$ 37 g differed among cycle phases, being highest in peak phase, followed by increase and decrease phases (Fig. 4). Females showed a pattern of phase-related changes in body mass similar to males (Fig. S2).

### Factors related to body mass

**Figure 5:**
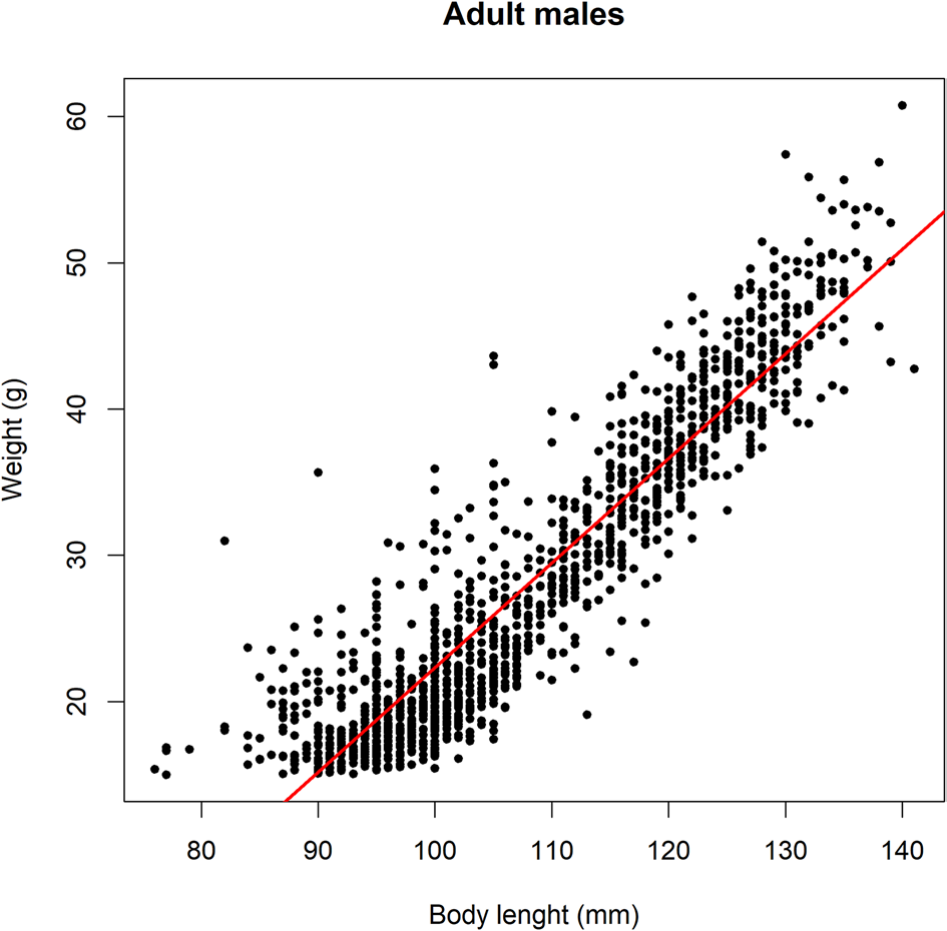
Scatterplot showing relationship between body mass and body length (snout-to-tail length) for all the common vole males trapped since March 2014 to November 2016. Regression line (red) is depicted (*R*^*2*^ = 0.85, *p* < 0.001).

Body mass was highly correlated with body length in adult males (R^2^ = 0.85; F_1 and 1311_ = 7415, p < 0.001) (Fig. 5), suggesting that differences in body mass are related to overall somatic growth, with heavier males being also structurally larger.

**Figure 6:**
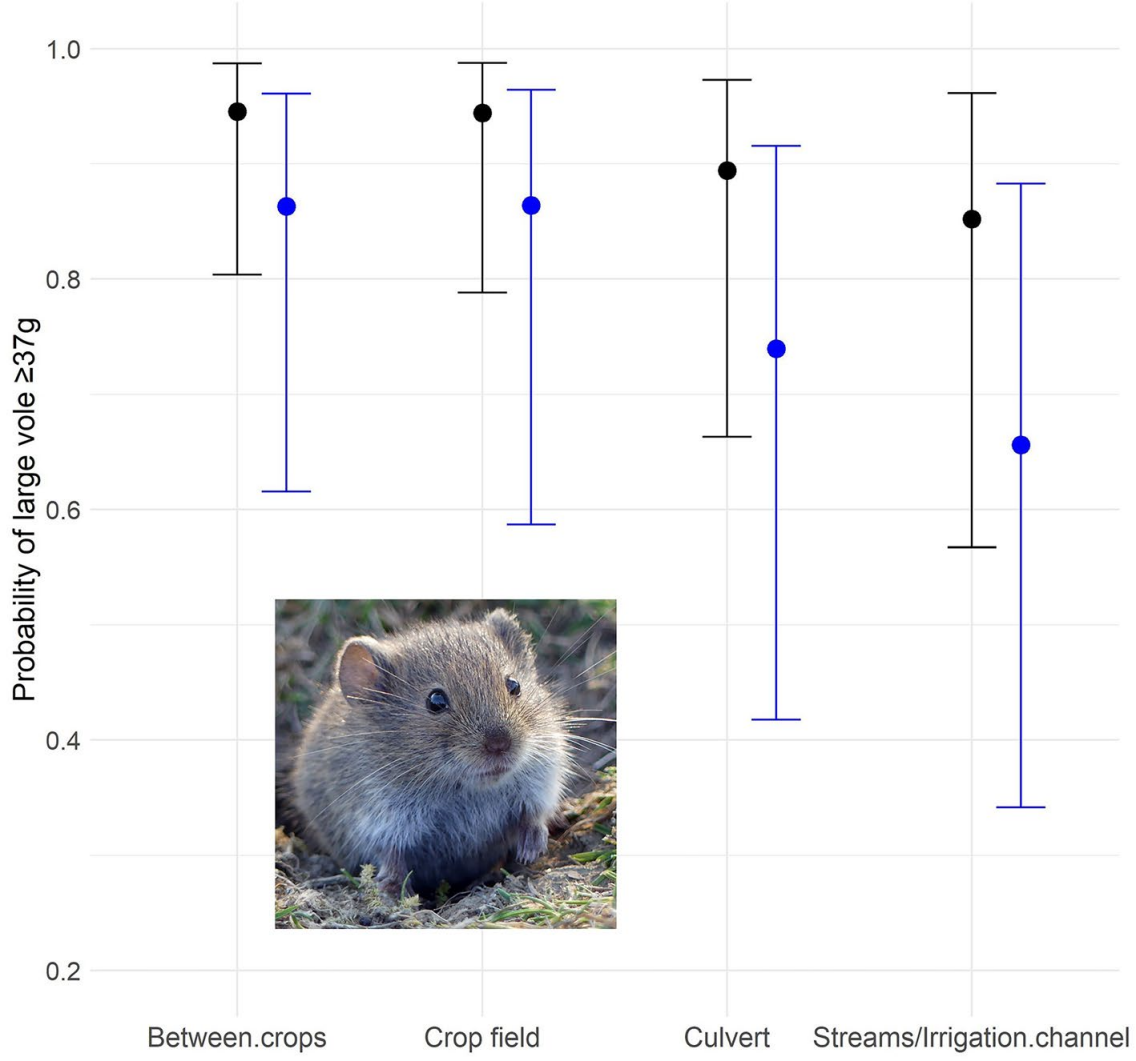
Probability of occurrence of large-size vole (body mass $\ge$ 37 g) during the cycle phases *increase* (black) and *peak* (blue) in different types of habitats. Mean (point) ± 95% confidence intervals (error bars) of the predictions of the GLMM (full model) at the population-level are shown. To estimate the predictions of the GLMM, all other fixed variables were held constant: continuous variables at their values means (i.e. vegetation height and cover ) and categorical variables at the levels “scrotal” (testes) and “Boada” (locality). Photo by *Gudrun Treiber*.

The GLMM fitted including the four-level habitat variable had a higher explanatory power (R^2^ = 0.74) than the model including the two-level habitat variable (R^2^ = 0.63). The first GLMM model showed that, as expected, trapping period had a significant effect on the probability of vole being heavier than 37 g (model AIC with *trapping period* vs AIC without: 457 vs 481; LRT: ${\chi }_{4}^{2}$=32.3, *p* < 0.0001). In March 2014 it was more likely to find a vole male weighing > 37 g than in June (β = − 1.11 ± 0.37; *p* = 0.003) and November 2014 (β = − 4.16 ± 1.11; *p* = 0.0002); yet no differences were found with March 2015 (β = − 3.45 ± 3.52; *p* = 0.990; likely by small sample size in this period) and November 2016 (β = − 2.40 ± 1.45; *p* = 0.100) (Table3). Weighing > 37 g was more likely for males with scrotal testes than without (β = − 3.85 ± 0.30; *p* < 0.0001) (model AIC with *scrotal testes vs* AIC without: 457 vs 698; LRT: ${\chi }_{1}^{2}$=246.6, *p* < 0.0001). Type of habitat where voles were captured significantly explained the presence of voles weighing > 37 g (model AIC with *habitat vs* AIC without: 457 vs 460; LRT: ${\chi }_{3}^{2}$=8.43, *p* = 0.038). Voles captured in margins between crops had higher probability of weighing > 37 g than voles captured in culverts (β = − 0.73 ± 0.38; *p* = 0.044) and in margins adjacent to streams and irrigation channels (β = − 1.14 ± 0.42; *p* = 0.006). The occurrence of heavier vole males (> 37 g) captured inside crop fields did not differ from those in between-crop margins and culverts, but it was significantly higher in crop fields than in margins adjacent to streams and irrigation channels (β = − 1.06 ± 0.52; *p* = 0.043) (Table 3**; **Fig. 6). There was no statistical evidence of an effect of locality, vegetation height or cover (LRT: ${\chi }_{1}^{2}$=0.01–0.77, *p* = 0.38–0.97) (Table 3).

**Table 3:** Results of the GLMM explaining occurrence of vole body mass $\ge$ 37 g (i.e. Chitty effect) including either the variable *trapping periods* (above table) or the variable *cycle phases* (below table).

	Estimate	Std. error	z value	Pr( >|z|)
Model with trapping periods				
(Intercept)	− 2.28	0.63	− 3.63	< 0.0001
Trapping period June 2014	− 1.11	0.37	− 2.99	0.003
Trapping period November 2014	− 4.16	1.11	− 3.74	0.0002
Trapping period March 2015	− 34.85	35,150,000	0.00	0.99
Trapping period July 2016	− 2.40	1.45	− 1.65	0.10
Reproductive status	3.85	0.30	12.82	< 0.0001
Habitat_culvert	0.37	0.38	0.98	0.33
Habitat_between.crops	1.14	0.42	2.75	0.006
Habitat_crop.field	1.06	0.52	2.03	0.04
Vegetation height	0.00	0.01	0.03	0.97
Vegetation cover	0.00	0.00	0.88	0.38
Locality	0.08	0.30	0.26	0.79
Model with cycle phases				
(Intercept)	− 6.41	1.16	− 5.51	< 0.0001
Reproductive status	3.83	0.30	12.86	< 0.0001
Phase_increase	4.13	1.11	3.72	0.0002
Phase_low	− 14.44	10,940	0.00	0.99
Phase_peak	3.02	1.07	2.84	0.005
Habitat_Culvert	0.33	0.37	0.87	0.38
Habitat_Between.crops	1.12	0.41	2.71	0.007
Habitat_Crop.field	1.05	0.52	2.02	0.04
Vegetation height	0.00	0.01	− 0.03	0.97
Vegetation cover	0.00	0.00	0.87	0.38
Locality	0.11	0.30	0.37	0.71

The GLMM including cycle phases explained 64% of the variance (R^2^ = 0.64) and was consistent with results of GLMM which included trapping period. There was statistical evidence supporting a significant effect of the phase of the population cycle on the probability of vole being heavier than 37 g (model AIC with *cycle phase vs* AIC without: 456 vs 481; LRT: ${\chi }_{3}^{2}$=31.4, *p* < 0.0001). The occurrence of large vole males was most likely in increase and peak phases than in decrease and low ones. Increase phases had a higher probability of occurrence of large voles than in peak phase (β = 1.11 ± 0.37; *p* = 0.003, Table 3**, **Fig. 6). As in the previous model, reproductive status and type of habitat showed statistical support of an effect significant (LRT: ${\chi }_{1}^{2}$=242, *p* < 0.0001 and ${\chi }_{3}^{2}$=8.29, *p* < 0.040, respectively). No significant effect of locality, vegetation height or cover were detected (Table 3). Results with the alternative variable cycle phases, which included July 2016 as increase phase, did not change the results (Table S1).

AIC-based model selection showed that one of the variables cycle phases and reproductive status was included in all the 16 models within 10 AIC units from the top model (sum of weights = 1). The rest of variables: habitat type (sum of weights = 0.74), vegetation cover (0.34), locality (0.29) and vegetation height (0.28) were each included in 8 of the 16 models (Table S2).

Results obtained analyzing probability of large voles, 90th percentile ($\ge$ 43.7 g) were qualitatively equivalent to those of 75th percentile (results not shown).

## Discussion

In this study, we addressed phase-related changes of body mass in cyclic populations of the Iberian common vole to determine the existence of what is long-known as Chitty effect and examined possible underlying factors of this common phenomenon in rodents. This (epi)phenomenon is usually defined as either: (i) the appearance during high population density phases of individuals 20%-30% heavier than in low density phases^11^ or (ii) the population average weight being higher in the increase and peak phases of the population cycle^9,52^. In our studied population, average body mass was higher in March 2014, June 2014 and July 2016, i.e. during growing or peak phases (Figs. 3, 4 and 6) and, in these phases, percentages of large-sized voles ($\ge$ 37 g) were much higher (24–38%) than in other phases (0%− 7.6%, low and decrease phases; March 2015, November 2014 and November 2016 in our study). The heavier adult males captured during increase and peak phases ranged from 54 to 61 g, and population means ranged from 27 to 35 g (Table 1**, **Figs. 3 and 4). Therefore, during growing or peak phases voles were more than 30% heavier than the average weight of adult males captured during low phases of the population cycle (means 19–21.7 g). In the decline or low phase of the first cycle (November 2014 and March 2015), males in the 90th percentile of body size distribution completely disappeared (> 43.7 g), and in November 2016 (start of decline phase, see^40^), only one male was heavier than 44 g. Our results coincide with other studies carried out on rodent populations showing Chitty effect, particularly with those showing large increase in body size^8,12,51,52,56–59^. Therefore, this work shows for first time empirical evidence of presence of marked Chitty effect in males (and probably females too, Fig S2) of cyclic common vole populations at the southern edge of the range of the species, in a Mediterranean area. Thus, it seems that Chitty effect is a common trait of cyclic rodent populations from arctic to Mediterranean latitudes, both in America and Europe.

The study period included two population peaks with markedly different maximal densities (2014 and 2016; Fig. 2**, **Fig. A1; see also^40,60^). Maximal abundance detected in the peak of 2016 was less than half that in 2014 (Fig. 2)^40^, likely due to population growth being curtailed by an extended drought period starting from June 2016 and persisting until the next year^60^—an unfavorable climate scenario for a species heavily dependent on the presence of green vegetation (e.g.^36^). Supporting this view, in June 2014, the frequency distribution histogram fitted well a bimodal distribution which suggest the presence of two different size groups within the population, being reflected in the highest coefficient of variation observed in the whole study period (Table 1). These two groups would be different cohorts: bigger and older males, and younger and smaller ones recruiting into the population at peak time. In contrast, during the peak of 2016 we did not find any bimodality in the distribution of body size (Fig. 3), probably due to adverse weather conditions reducing productivity and recruitment of large-sized individual. However, Chitty effect was detected in a similar way in both peak years, what suggests that this population process is quite independent of prevailing vole density, but it would be more related to phase of the cycle, independently of maximal densities reached. In fact, our analysis of factors promoting the appearance of large size individuals pointed to trapping session and phase of cycle as the main explanatory factors (Table 3). Our results are coincident with recent results in Russia showing that Chitty effect disappears in vole populations losing cyclicity, so it seems to be a population syndrome clearly associated to cycles^61^, but see^12^.

Large-size males appeared already during March 2014, before reaching maximal densities in June, which suggest that Chitty effect starts to appear at least during the final increase phase of the cycle. Unfortunately, we do not have data for spring 2016, so this result must be confirmed with additional research. If confirmed in upcoming high-density phases, the appearance of large males in the population could work as a reliable early-warning signal of an approaching population outbreak, before reaching maximal densities. This could be highly useful for managers, given that common vole population outbreaks in NW Spain are quite irregular, both in the period elapsed between population outbreaks [peaks reported every 2–10 years, depending on prevailing weather conditions^6,35^, the area affected (apparently regional-scale outbreaks every 5 years on average, but peaks every 2–3 years in Tierra de Campos area), or maximal densities reached (e.g. maximal densities in the peak of 2016 smaller than those in the growing phase of spring 2014).

Several hypotheses have been proposed to explain the existence of Chitty effect (Table S3). Experimental work would be necessary to fully refute or support any of those hypotheses, but our observational results can be useful providing some insights about the plausibility of some of them.

The high correlation found between male body mass and body length (Fig. 5), supports that changes in the average body mass across trapping periods (Fig. 4) were mainly due to true changes in body size, such as the skeletal structure, and not merely to the amount of fat and muscle with respect to structural size (body condition). Thus, the hypothesis considering that Chitty effect is more an effect of improved condition instead of larger structural size would not be supported by our results^52^ (Table S3).

The presence of large-size males in both phases of increase and peak may respond to a period during which the population experienced increased food availability and higher survival rates. In other studies on rodents with cyclical population dynamics, it has been proposed that the presence of individuals with higher weight during periods of demographic growth is related to increased survival^58,62,63^. Under laboratory conditions, with unlimited food, the common vole can continue to grow up to 10 months of age, reaching an average weight of 45 g^64^. However, such individuals are not always found in nature because they tend to die before completing their growth due to insufficient food availability or predation. Under especially favorable conditions, such as the beginning of a strong demographic increase^23^, voles could experience access to more food and higher survival leading to a larger body size, as they have a longer growth period^58^ what would promote cohorts with larger voles. These large-size vole cohorts would coexist alongside smaller sized groups which would have faced worse environmental conditions. Our results suggest, however, that Chitty effect developed in a similar way during a high-density peak (associated to good environmental conditions) and during a lower-density peak in a year with poorer environmental conditions (drought since June 2016). Nonetheless, the drought could have affected the population when large-sized voles were already present, as in July 2016 the population showed a high average body mass, and thus adverse weather conditions would have affected rather the productivity and recruitment of small-size individuals (see above). Further evidence needs to be gathered to ascertain the role of prolonged favorable environmental conditions in explaining large-sized voles (e.g. ^12,24,48^) (Table S3).

Furthermore, we found that reproductive status also contributed to explain the appearance of large-size males (Table 3), so large size males tended to be reproductively active. Indeed, 59% of large-size males (> 37 g; N = 330) were reproductively active *versus* 15% for small-size males (< 37 g, N = 987). Thus, we found no clear evidence supporting breeding suppression in large-size males, a necessary condition for some of the hypotheses proposed whereby the supposed energy savings from reproduction are diverted towards somatic growth^9,65^ (Table S3). Indeed, the reduction or suppression of reproduction is rather linked to thermal conditions, a recognized energy-demanding factor^66^. Interestingly, recent work with non-cyclic rodents has shown a density-dependent increase in body size during periods of growth in population density^67,68^. Petrova et al.^68^ concluded that in year with good environmental conditions large-size individuals had higher productivity, reflected in subsequent high population density. That is, large individuals contributed most to reproduction causing population growth, contrary to some hypotheses proposed to explain Chitty effect in cyclic rodents (Table S3).

Finally, we found that the probability of detecting large-size males depended on the kind of habitat prospected (Fig. 6). Optimal crops (mainly alfalfa) and margins between crop fields had higher probability of occurrence of large-sized voles than the other two kind of field margins considered (culverts and edge of streams or irrigations channels). To our knowledge, this is the first time that Chitty effect is statistically related to the type of habitat. Higher occurrence of large-sized voles in the optimal habitat for voles in agricultural landscapes, alfalfa fields, could be interpreted as a consequence of high-quality and abundant food promoting enhanced growth, but this would not be the case for between-crops margins, which are narrow strips of sparse herbaceous vegetation^42^. In fact, the margins holding apparently better habitat for voles were those adjacent to streams or channels that tended to be wide strips of dense and tall herbaceous vegetation^40^, but those margins just had the lowest probability to hold large-sized voles (Fig. 6). The main specialist ground predator of voles, the weasel (*Mustela nivalis*), is common in the study area^69,70^ and its populations suffer abundance cycles coupled to those of voles, with a slight delay^6^. In this kind of agricultural landscapes, weasels use almost exclusively undisturbed and well vegetated field margins, probably because they are the only habitat with long-term stability (e. g. unexposed to harvesting, ploughing or other perturbations associated to crop management) and well developed vegetation (in contrast with between-fields margins), providing protection from aerial predators, so their home ranges have a reticular shape and crop fields are very rarely used^71^. Thus, the habitats with the highest probability of holding large-sized voles (crops and poorly vegetated between-crops margins) were the ones less used by weasels, while the optimal habitat for weasels, wide and well vegetated strips in the edge of streams and channels, had the lowest probability to hold large-sized voles. Thus, our results seem to fit a major prediction of the size selective predation hypothesis^72^ (Table S3) whereby large-sized voles were less abundant in optimal habitats for weasels, that would remove from vole populations those larger individuals more easily than smaller ones. Moreover, in a vole population subjected to predation by weasels, reaching a certain age and thus large body size would be less likely than in an environment without predation or with a more relaxed predation pressure. This could occur due to either a direct effect (mortality) or an indirect effect (landscape of fear, where individuals forage less and grow less). Interestingly, Santamaría et al.^42^ found in the same vole population that, at landscape scale, a larger area covered by streams was associated to lower abundance of common vole burrows. Planillo et al.^40^, also for the same vole population, reported that vegetation structure was a major factor explaining vole abundance in field edges during peak phase, so less disturbed margins (better for weasels), which in our study area was related to low cover but high vegetation, had lower vole density at peak time. Those results were associated to the potential regulatory effect of vole abundance caused by weasel predation in the optimal habitat for the predator. Taken together, all these results would support that predation by weasels in our study area is an important regulatory factor, both of overall vole abundance and, more specifically, of occurrence of large-sized voles. On the other hand, our localities differed in terms of avian predation pressure (assuming the installment of nest boxes for raptors had the intended effect). However, we did not find any differences between localities in the probability of large-sized vole, likely because aerial raptors do not exert any selection on vole size, in contrast to weasels^72^.

We encountered some limitations in our study. During the spring of 2016, we lack data on voles, which prevented us from having two complete population cycles that would have allowed us to strengthen some of our results and conclusions. Nonetheless, the effect of period and cycle phase was clear and robust in our analyses. Although we sampled the vole population regularly (3–5 months), more frequent sampling (e.g. every 2 months) would have been more appropriate to determine more accurately the patterns of change in body mass in the population. Additionally, we were unable to measure the age of the individuals, which prevented us from determining whether the larger voles resulted from a prolonged growth period, as supported by some of the hypotheses regarding the Chitty effect (Table S3). Future studies on the topic would benefit from determining the age of the individuals for elucidating whether large-sized voles are the older ones. Additionally, measuring the effect of weasel predation pressure on the abundance of large-sized voles would enhance our understating of the Chitty effect.

As a corollary, we have demonstrated that the occurrence of large-sized vole within the common vole population at the southern range edge significantly increased in reproductive males during the increase and peak phases, a pattern which was moderated by habitat. Our findings suggest that predation by weasel may mediate the relationship we observed between large-sized vole and habitat. Any action promoting higher density and/or stability of weasel populations may a be a crucial element for an environmentally friendly, ecologically-based vole control management program. As a first step, promoting the conservation or development of wide and well vegetated undisturbed field edges can be an easy start for such strategy (see^40^).

## Supplementary Information

Supplementary Information.

## Data Availability

The datasets used and/or analysed during the current study are available from the corresponding author on reasonable request.
