# Author Correction: Habitat type modulates sharp body mass oscillations in cyclic common vole populations

**DOI:** 10.1038/s41598-024-76122-5

**Published:** 2024-10-24

**Authors:** Pedro P. Olea, Noelia de Diego, Jesús T. García, Javier Viñuela

**Affiliations:** 1 Terrestrial Ecology Group (TEG), Departamento de Ecología, Facultad de Ciencias, Universidad Autónoma de Madrid (UAM), 28049 Madrid, Spain; 2 Centro de Investigación en Biodiversidad y Cambio Global (CIBC‑UAM), Universidad Autónoma de Madrid, 28049 Madrid, Spain; 3 Game and Wildlife Management Group, Institute for Game and Wildlife Research (IREC, UCLM-CSIC-JCCM), Ciudad Real, Spain

Correction to: *Scientific Reports* 10.1038/s41598-024-62687-8, published online 26 May 2024

In the original version of this Article, the Funding section was incomplete.

“This study was funded by I + D National Plan Projects of the Spanish Ministry of Economy, Industry and Competitiveness (TOPILLAZO-CGL2011-30274 and MOVITOPI-CGL2015-71255-P, co-funded by European Regional Development Fund FEDER, EU), Fundación BBVA Research Project TOPIGEPLA (2014 call) and MAPAMA/TRAGSATEC to GREFA (biological control program). Researchers at TEG-UAM were also funded by REMEDINAL TE-CM Research Network (P2018/EMT4338). Noelia de Diego was supported by a master thesis grant “Fomento de la Investigación” funded by Universidad Autónoma de Madrid.”

now reads

“This study was funded by I + D National Plan Projects of the Spanish Ministry of Economy, Industry and Competitiveness (TOPILLAZO-CGL2011-30274 and MOVITOPI-CGL2015-71255-P, co-funded by European Regional Development Fund FEDER, EU), Fundación BBVA Research Project TOPIGEPLA (2014 call) and MAPAMA/TRAGSATEC to GREFA (biological control program). This study has also benefited from the support of Research Project ref. 022-GRIN-34462, funded by the University of Castilla-La Mancha and the Fondo Europeo de Desarrollo Regional (FEDER). Researchers at TEG-UAM were also funded by REMEDINAL TE-CM Research Network (P2018/EMT4338). Noelia de Diego was supported by a master thesis grant “Fomento de la Investigación” funded by Universidad Autónoma de Madrid.”

The original Article has been corrected.

