# Supplementary material for: Habitat type modulates sharp body mass oscillations in cyclic common vole populations
Source: Sci Rep. 2024 May 26;14:12013. doi: 10.1038/s41598-024-62687-8 (PMC11128438; doi:10.1038/s41598-024-62687-8)
Supplement: Supplementary file 1 — Supplementary Information. [file 41598_2024_62687_MOESM1_ESM.pdf]

ESM (electronic supplementary material) –

Habitat type modulates phase-dependent body mass in cyclic vole populations

Pedro P. Olea[1,2*], Noelia de Diego[3], Jesús T. García[3], Javier Viñuela[3]

[1]Terrestrial Ecology Group (TEG), Departamento de Ecología, Facultad de Ciencias, Universidad Autónoma de Madrid (UAM), 28049, Madrid, Spain.
[2]Centro de Investigación en Biodiversidad y Cambio Global (CIBC-UAM), Universidad Autónoma de Madrid, 28049, Madrid, Spain.
[3]Game and Wildlife Management Group, Institute for Game and Wildlife Research (IREC, UCLM-CSIC-JCCM), Ciudad Real, Spain

Corresponding author: pedrop.olea@uam.es

Table S1. Results of the GLMM explaining occurrence of vole body mass ≥37g (i.e. Chitty effect) including the variable cycle phases in which July 2016 was considered as increase phase (below table).

Model with cycle phases				
	Estimate	Std. Error	z value	Pr(>|z|)
(Intercept)	-2.63	1.14	-2.30	0.02
Reproductive status	-3.79	0.30	-12.84	<0.0001
Phase_increase	3.99	1.11	3.61	0.0003
Phase_low	-15.83	21450	-0.001	1.00
Phase_peak	3.03	1.07	2.85	0.004
Habitat_Culvert	0.31	0.37	0.83	0.40
Habitat_Between.crops	1.13	0.41	2.75	0.006
Habitat_Crop.field	1.04	0.52	2.01	0.045
Vegetation height	-0.0001	0.006	-0.020	0.98
Vegetation cover	0.004	0.005	0.84	0.40
Locality	0.18	0.29	0.62	0.54

Table S2. Ranking of **models on the probability of occurrence of large vole (body mass ≥37g)** (i.e. Chitty effect) ordered according to AICc and weight of the models. “+”: denotes a categorical variable included in the model; blank cell denotes that the variable is not included into model; the coefficients of continous variables and estimates of loglik, AICc, delta AICc and weight of each model are shown. Only models within 10 AICc units from the best model are shown.

Intercept	Vegetation height	Vegetation Cover	Reproductive status	Cycle Phases	Type of habitat	Locality	df	logLik	AICc	delta	weight
-6.071			+	+	+		9	-216.6	451.4	0.0	0.242
-6.366		0.005	+	+	+		10	-216.0	452.4	1.0	0.145
-6.175			+	+	+	+	10	-216.4	453.1	1.7	0.103
-6.101	0.001		+	+	+		10	-216.5	453.4	2.0	0.088
-5.523			+	+			6	-220.7	453.6	2.2	0.081
-6.410		0.004	+	+	+	+	11	-216.0	454.3	2.9	0.056
-6.360	-0.001	0.005	+	+	+		11	-216.0	454.4	3.1	0.052
-5.371		-0.004	+	+			7	-220.3	454.7	3.4	0.045
-5.463	-0.004		+	+			7	-220.4	454.9	3.5	0.042
-6.220	0.001		+	+	+	+	11	-216.3	455.1	3.7	0.038
-5.548			+	+		+	7	-220.7	455.6	4.2	0.030
-6.407	0.000	0.004	+	+	+	+	12	-216.0	456.4	5.0	0.020
-5.374	-0.003	-0.003	+	+			8	-220.2	456.5	5.2	0.018
-5.415		-0.004	+	+		+	8	-220.2	456.6	5.3	0.017
-5.484	-0.004		+	+		+	8	-220.3	456.9	5.5	0.015
-5.409	-0.003	-0.003	+	+		+	9	-220.1	458.5	7.1	0.007
-3.273			+		+	+	7	-232.6	479.4	28.1	0.000

Table S3. Summary of hypothesis proposed to explain Chitty effect in cyclic rodent populations.

Reference	***Species***	***Location***	***Conclusions***
1	*California vole (Microtus californicus)*<br>*Meadow vole (Microtus pennsilvanicus)*<br>*Prairie vole (Microtus ochrogaster)*<br>*Townsend's vole (Microtus townsendii)*	*USA*	*Bigger body size is* ***a genetic trait****. 2 opposite hypotheses:*<br>*Being larger as an adult is an adaptative advantage in* ***increasing populations,*** *but are not well suited to live at a high population density.*<br>*Larger adults are more aggressive and positively selected at* ***high population density****, but they don't have an advantage in the increasing phases.*
2	*Field vole (Microtus agrestis)*	*Sweden*	*Larger individuals have a competitive advantage in* ***digesting food more efficiently****, due to their larger digestive system.*
3	*California vole (Microtus californicus)*	*California, USA*	*Extra-large males seem to be the result of prolonged periods favorable to* ***growth and survival****. They* ***do not have an enhanced fitness*** *under conditions of rapid population growth and high densities.*
4	*Field vole (Microtus agrestis)*<br>*Bank vole (Myodes glareolus)*	*Sweden*	*Cyclic and noncyclic populations of voles demonstrated an initial dimorphism or polymorphism in size. Large-sized animals show continuous growth and can be distinguished early in life. There is evidently a* ***selection against larger voles at a young age in less benign environments****.*

5	*Bank vole (Myodes glareolus)*	*France*	*Larger body weight is the result of a **higher survival rate** selecting for **lower reproductive effort** and a **higher somatic allocation. Lower predation** may contribute to the higher survival rate.*
6	*Several vole species*	*Theoretical*	***Reproduction is suppressed** in animals born or raised in the later part of the increase phase by environmental factors. The **surplus energy** will allow continuous **growth**. Animals grow to a larger size as a population enters the peak density phase, causing an increase in the average body*
7	*Sibling vole (Microtus rossiaemeridionalis)*<br><br>*Field vole (Microtus agrestis)*<br><br>*Bank vole (Myodes glareolus)*	*Finland*	*Changes in body condition rather than mere size (body length). It is suggested that previous density of voles mainly affects the quality of voles indirectly through changes in the biotic environment, and that the proximate cause behind the Chitty effect is the **combined effect of changes in predation pressure and availability of food.***
8	*Sibling vole (Microtus rossiaemeridionalis)*<br><br>*Field vole (Microtus agrestis)*	*Finland*	*Negative association between the mean body size of voles and weasel abundance was found in field observations. Laboratory experiments showed that smaller voles can escape from weasel predation, because they fit in holes too narrow for weasels. **Size selective predation** might be an important cause of the observed smaller voles in the decline phase of cycles.*
9	*Common vole (Microtus arvalis)*	*Czech Republic*	*Changes in **food supply affect individual development**. Voles reach a lower body size in suboptimal habitats.*
10	*Bank vole (Myodes glareolus)*<br><br>*Grey-sided vole (Myodes rufocanus)*	*Norway*	- *Bank voles: Body weight is positively related to population density, due to a higher rate of survival at higher densities resulting from individual **allocation of resources from reproduction to survival and growth.***

			- *Grey-sided voles: A negative delayed density dependence for body weight was found. The **environmental conditions in the preceding year determine current body weight.***
11	*Field vole (Microtus agrestis)*	*England*	*Although voles reach highest asymptotic weights in the peak phase and lowest asymptotes during the crash, initial growth rates were not significantly different. This suggests that voles attain larger body size during the peak phase as a result of **growing for longer.***
12	*Meadow vole (Microtus pennsilvanicus)*	*Virginia, USA*	*The large body masses observed in some voles were due more to **long field lives** than to unusually high rates of body growth.*
13	*Prairie vole (Microtus ochrogaster)*	*Illinois, USA*	***Higher somatic growth** in increase and peak phases of the population cycle.*
14	*Bank vole (Myodes glareolus)*	*Finland*	*Voles from different cycle phases kept the same growth patterns when relocated to the same environment. Body size traits might be strongly influenced by **heredity or early life conditions,** rather than that of current juvenile and adult environmental conditions.*

References:

1. Boonstra, R., Krebs, C. J. (1979). Viability of large- and small-sized adults in fluctuating vole populations. Ecology 3: 567-573.

2. Hansson, L., Jaarola M. (1989). Body size related to cyclicity in microtines: dominance behaviour or digestive efficiency? Oikos 55:356–364.

3. Lidicker Jr, W. Z., Ostfeld, R.S. 1991. Extra-large body mass in California voles: causes and fitness. Oikos 61: 108-121.

4. Hansson, L. (1995). Size dimorphism in microtine populations: characteristics of growth and selection against large-sized individuals. Journal of Mammalogy 76(3): 867-872.

5.Yoccoz, N.G. and Mesnager, S. 1998. Are alpine bank voles larger and more sexually dimorphic because adults survive better? Oikos 82:85–98

6. Oli, M. K. (1999). The Chitty Effect: A Consequence of Dynamic Energy Allocation in a Fluctuating Environment. *Theoretical Population Biology*, *56*(3), 293–300. https://doi.org/10.1006/tpbi.1999.1427

7. Norrdahl, K., Korpimäki, E. (2002a). Changes in individual quality during a 3-year population cycle of voles. Oecologia 130(2): 239-249.

8. Sundell, J., Norrdahl, K. Body size-dependent refuges in voles: an alternative explanation of the Chitty effect. Ann. Zool. Fennici: 325-333 (2002).

9. Jánová, E., Heroldová, M., Bryja, J. (2008). Conspicuous demographic and individual changes in a population of the common vole in a set-aside alfalfa field. Annales Zoologici Fennici 45(1): 39-54.

10. Johannesen, E., Andreassen, H.P. (2008). Density-dependent variation in body mass of voles. Acta Theriol. 53:169–173

11. Burthe, S.J., Lambin, X., Telfer, S., Douglas, A., Beldomenico, P., Smith, A. & Begon, M. (2010). Individual growth rates in natural field vole, Microtus agrestis, populations exhibiting cyclic population dynamics. Oecologia 162(3): 653-661.

12. Longtin, S.B. and Rose, R.K., 2012. Unusually high body mass in Virginia meadow voles. Journal of Mammalogy 93(3): 743-750.

13. van Benthem, K. J., Froy, H., Coulson, T., Getz, L. L., Oli, M. K., & Ozgul, A. (2017). Trait-demography relationships underlying small mammal population fluctuations. *Journal of Animal Ecology*, *86*(2), 348–358. https://doi.org/10.1111/1365-2656.12627.

14. Sundell, J., Ylönen, H., & Haapakoski, M. (2019). Do phase-dependent life history traits in cyclic voles persist in a common environment? *Oecologia*, *190*(2), 399–410. https://doi.org/10.1007/s00442-019-04410-3.

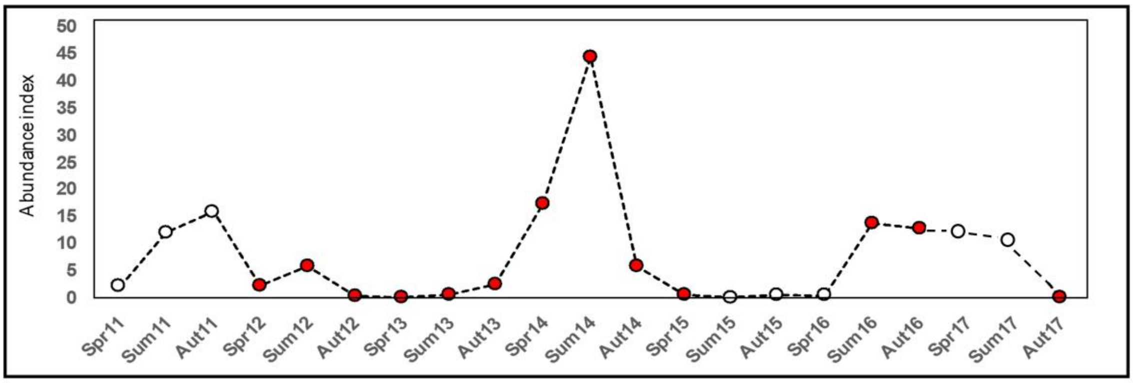

Figure S1. Long-term demographic fluctuations at regional scale in our study area (Palencia). Red points represent abundances (vole captures/100 traps-night) based on our own samplings, and white points represent abundances extracted from other published studies in the region (15-17).

Referencias:

15. Paz, A. 2021 Aplicaciones del control biológico mediante aves rapaces como herramienta para prevenir plagas agrícolas de topillo campesino (*Microtus arvalis*). Doctoral Thesis. Universidad de Alcalá.

16. Mougeot, F., Lambin, X., Rodríguez-Pastor, R., Romairone, J., Luque-Larena, J.J., 2019. Numerical response of a mammalian specialist predator to multiple prey dynamics in Mediterranean farmlands. Ecology 100, 1–14. https://doi.org/10.1002/ecy.2776.

17. Rodríguez-Pastor, R., Luque-Larena, J. J., Lambin, X., & Mougeot, F. (2016). "Living on the edge": The role of field margins for common vole (Microtus arvalis) populations in recently colonised Mediterranean farmland. *Agriculture, Ecosystems & Environment*, *231*, 206–217. https://doi.org/10.1016/j.agee.2016.06.041

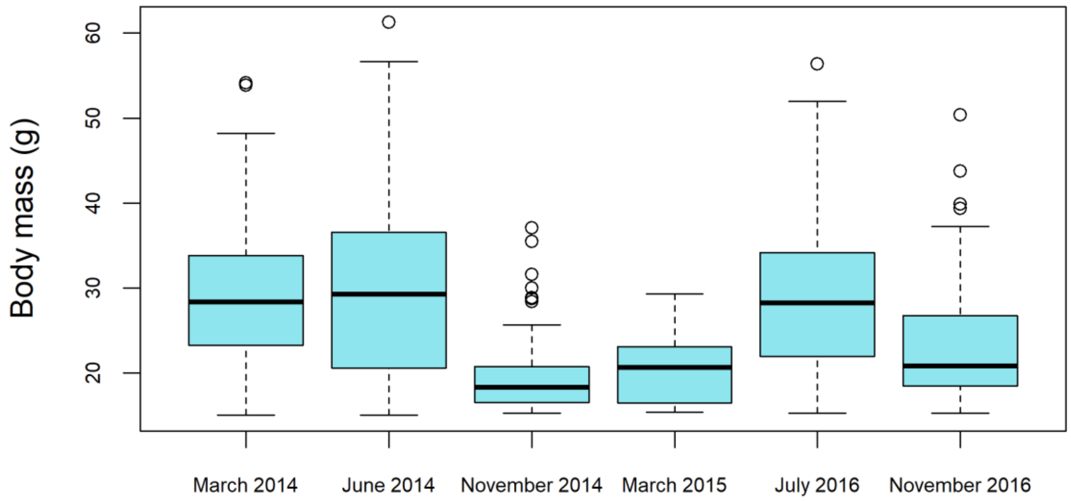

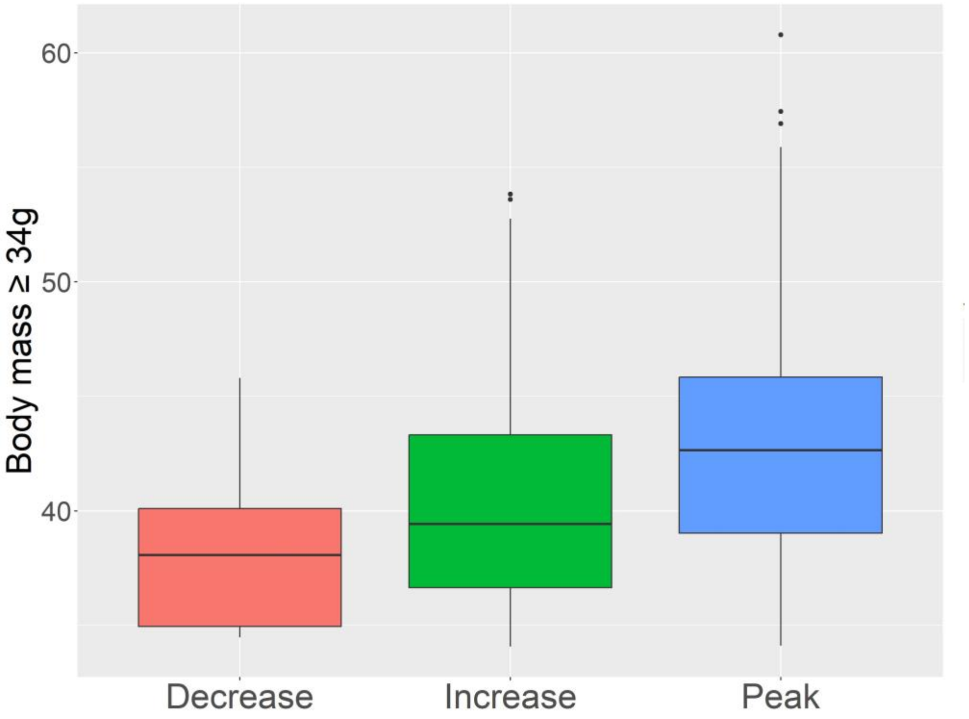

Figure S2. Boxplots for body mass of adult **female** voles trapped in each period (above) and for body mass ≥34g according to the cycle phases (below). Boxes represent 75% of data, and median is depicted as a black line. The length of the whiskers shows values from the 75th percentile up to 1.5 × IQR (upper whisker) and from the 25th percentile down to -1.5 × IQR (lower wisker). Pattern of body mass shown by females (plot above) was like males; yet somewhat difference can be observed in summer 2014 and 2016 in which medians of females were higher than males, likely due to a higher prevalence of pregnant females.
